# Cancer Screening Prevalence and Associated Factors Among US Adults

**DOI:** 10.5888/pcd19.220063

**Published:** 2022-04-21

**Authors:** Zhen-qiang Ma, Lisa C. Richardson

**Affiliations:** 1 Pennsylvania Department of Health, Harrisburg, Pennsylvania; 2 Centers for Disease Control and Prevention, Atlanta, Georgia

Cancer is the second leading cause of death in the US, exceeded only by heart disease. In 2018, 1,708,921 people were newly diagnosed and 599,265 people died of cancer (1). Although age-adjusted cancer incidence decreased 9.5% over the past 20 years, from 481.7 per 100,000 in 2009 to 435.8 per 100,000 in 2018, the number of people diagnosed with cancer increased, from 1,292,222 in 2009 to 1,708,921 in 2018 (1,2). The estimated national expenditure for cancer care in the US rose from $190.2 billion in 2015 to $208.9 billion in 2020, a 10% increase mainly due to the aging and growth of the US population (3,4). Costs will likely increase in future years as the population grows and ages and new and often more expensive treatments are adopted as standards of care.

Approximately 30% to 50% of cancers diagnosed today could be prevented by reducing exposure to tobacco smoke and other environmental carcinogens, maintaining healthy body weight, and receiving recommended cancer screenings and vaccinations (5,6). Cancer screening, which is different from diagnostic testing, can detect cancer at early stages before symptoms occur, when it can be more successfully treated. In addition to early detection, screening can prevent colorectal and cervical cancers by identifying precancerous lesions that can be removed before they become cancer (7–9). Thus, understanding screening patterns and factors associated with screening will help public health policy makers and practitioners improve cancer prevention programs further by implementing evidence-based policies and practices (10,11). This special collection of articles from *Preventing Chronic Disease* presents research on determinants of cancer screening, public health practices that increase cancer screening uptake in specific populations, and cancer screening trends.

Screening is considered the primary factor in the steady decline in colorectal cancer incidence over the past decade (12). Richardson and colleagues used data from the Behavioral Risk Factor Surveillance System to present a GIS (geographic information system) snapshot of US states and the District of Columbia that displays the percentage of US adults who reported no screening for colorectal cancer (13). The overall percentage screened decreased from 27.4% in 2012 to 21.6% in 2020, a 5.8 percentage=point decrease that represents almost 4 million people. The average statewide percentage of adults aged 50 to 75 years who were not up to date with colorectal cancer screening in 2020 was 69.4% and ranged from 58.4% in California to 79.6% in Maine. Twenty-two states did not meet the Healthy People 2020 objective of 70.5% of population screened for colorectal cancer. And most adults not up to date with screening had never been screened. Future research on colorectal cancer screening could focus on population subgroups and on new outreach methods directed at the unscreened in those subgroups. Successful interventions could then be disseminated among other population subgroups.

Although overall age-adjusted cancer incidence has been stabilizing over the past several decades, Weir and colleagues used the age-period-cohort generalized linear model to predict that total cancer incidence in the US will increase approximately 50% from 2015 to 2050, from 1.5 million to 2.3 million (2). The largest increase in cancer incidence will occur in people aged 75 years or older; prevention and early detection do work in older populations (14). With the US population aging and age as a nonmodifiable risk factor for cancer, prevention programs can implement evidence-based risk-reduction strategies to reduce behavioral risk factors such as smoking, drinking, and exposure to environmental carcinogens and chronic conditions such as obesity and type 2 diabetes. Cancer screening could also be treated as a prevention priority to detect precancerous lesions that can be removed, thereby preventing cancer, and to detect cancers at early, treatable stages. State and local health departments could also use the age-period-cohort model to estimate their local cancer incidence in their respective state and local areas and develop actionable plans with innovative strategies to help residents change their behaviors by making healthy lifestyle choices, including increasing screening rates. State and local health departments can also use the model to evaluate cancer prevention program outcomes by comparing the time trends and differentials of cancer incidences with or without interventions.

Screening can prevent thousands of cancer deaths. Modern mammography programs can reduce breast cancer mortality by more than 40% (15–17). The over-50% decrease in cervical cancer incidence and mortality over the past 3 decades is largely due to screening with the Papanicolaou (Pap) test, which can detect cervical cancer at an early stage as well as precancerous abnormalities (9). With appropriate evaluation, follow-up, and treatment, survival for women diagnosed with precancerous cervical lesions is almost 100% (18). Sharma and colleagues used a model-based approach in a cohort of 50-year-old participants and estimated that 10,179 deaths from breast cancer, 27,166 from cervical cancer, and 74,740 from colorectal cancer could be prevented if current screening levels were maintained. In addition, an extra 1,300 deaths from breast cancer, 3,400 from cervical cancer, and 11,000 from colorectal cancer could be averted with an increase of 10 percentage points above current screening rates (19). However, even with its proven benefits and US Preventive Services Task Force (USPSTF) recommendations, cancer screening is still suboptimal. The median prevalence of women aged 50 to 74 years who had a mammogram within the past 2 years was about 78% in 2020 and varied substantially, from 66% to 87% among states, differing by race and ethnicity, household income, access to health care, age, and education level (20). However, in 2020 approximately 20% of women aged 21 to 65 years had not been screened for cervical cancer in the past 3 years (20). Moreover, the national median prevalence of people aged 50 to 75 years who have been screened for colorectal cancer per USPSTF recommendations remains less than 70% (13). Again, screening rates differ substantially by state, age group, race and ethnicity, access to health care, health insurance, household income, and education level (20).

Many factors could affect cancer screening behavior, including sociodemographic characteristics, screening cost, health insurance, education, income, travel distance to and location of screening sites, knowledge of the disease, patient and clinician attitudes, and availability of adequate health care facilities (1,15–17,21,22). Therefore, investigating factors influencing screening participation is crucial to creating and implementing population-based cancer screening programs. One such program is the National Breast and Cervical Cancer Early Detection Program (NBCCEDP; www.cdc.gov/cancer/nbccedp/), which was authorized under the Breast and Cervical Cancer Mortality Prevention Act of 1990. The program provides breast and cervical cancer screening and diagnostic services to low-income, underinsured, and uninsured women. NBCCEDP focuses on factors at the interpersonal, organizational, community, and policy levels that influence screening and has served more than 5.9 million women with more than 15.4 million breast and cervical cancer screenings since its inception in 1991. NBCCEDP has expanded and now funds 70 award recipients — all 50 states, the District of Columbia, Puerto Rico, 5 US Pacific Island territories, and 13 American Indian and Alaska Native tribes or tribal organizations. Such programs directed at medically underserved populations should be expanded throughout the country.

Benavidez and colleagues used 2018 BRFSS data to study women who met breast, cervical, and colorectal cancer screening consistent with USPSTF recommendations and found that screening disparities persisted among socioeconomically disadvantaged groups, especially low-income women and women without health insurance (23). They also found that Hispanic women had higher breast and cervical cancer screening prevalence but lower colorectal cancer screening prevalence than non-Hispanic White women. In addition, some racial and ethnic groups and rural populations are disproportionately affected by most cancers. Kruse-Diehr and colleagues compared colorectal cancer deaths in Black populations with White populations in the historically segregated and economically distressed Mississippi Delta. They reported that segregation affected Black and White populations differently. Deaths from colorectal cancer among Black people were higher in mildly and severely segregated urban counties than in moderately segregated counties. Segregation had no effect on colorectal cancer death rates among Black populations in rural counties and was not associated with death rates among White populations (24). Bhimla and colleagues evaluated factors related to colorectal cancer screening among populations of Asian descent by neighborhood ethnic density and psychosocial factors, including knowledge about colorectal cancer, self-efficacy about screening, and perceived barriers to screening behaviors. Their study found that Vietnamese and Filipino Americans had significantly lower screening rates than Korean Americans (25). They also showed that Asian Americans who lived in neighborhoods with high Asian ethnic density were unlikely to complete the colorectal cancer screening process. These findings suggest that the people providing health education to populations with low colorectal cancer screening prevalence could benefit from a better understanding of the cultural norms and beliefs of those populations. Research on cultural characteristics is warranted to understand better why screening differences exist among different racial and ethnic populations. One successful study funded by the Centers for Disease Control and Prevention (CDC) showed that designing interventions for breast and cervical cancer for Muslim women could facilitate screening (26).

In an analysis of a large federally qualified health center in central Texas, Zhan and colleagues found that colorectal cancer screening prevalence was low among people who lived more than 20 miles from a primary care clinic. On the other hand, they found that screening prevalence was high among people who visited their primary care provider regularly. They also used geospatial cluster analysis to identify clusters of patients not up to date with colorectal cancer screening (27).

A randomized clinical trial showed that 20% fewer lung cancer deaths occurred in a group that received an invitation to annual low-dose computed tomography (LDCT) screening compared with a group invited to receive annual chest x-rays (22). Rohatgi and colleagues completed a quantitative evaluation of geographic access to LDCT lung cancer screening in Missouri and Illinois. They reported that rural residents had significantly lower access to LDCT than urban residents (28).

Where a person lives can profoundly affect short- and long-term health (29). Much research into this relationship incorporates locality and geospatial analysis with mixed-model approaches, which can be adopted by state and local health departments by using patient data. Although some geospatial research was done at the county level because of data constraints, geospatial analysis could be further developed for small neighborhoods where homogeneity can be found at the subcounty level. To answer this need, CDC developed PLACES (www.cdc.gov/places/) with the support of the Robert Wood Johnson Foundation and the CDC Foundation. PLACES uses small area estimation methods to provide community estimates on health conditions, prevention, health risks, and health status down to the zip code tabulation area (30). The PLACES tool can help us better understand why the uptake of cancer screening did not reach Healthy People 2020 targets. These data also allow public health professionals to identify populations for implementing proven interventions.

The Community Preventive Services Task Force (*Community Guide*) provides many evidence-based findings and recommendations about cancer screening in community settings (31). These recommendations can be adopted and modified for specific localities and populations. Haverkamp and colleagues mailed a fecal immunochemical test (FIT) to the eligible population served by 3 health care facilities in Arizona operated by American Indian tribes. They found that direct mail to eligible tribe members with instructions and a follow-up telephone call and/or home visit improved the screening compliance rate significantly (32). Simply mailing the FIT test kit with instructions and a telephone call reminder to eligible patients with regular office visits increased the test kit return rate almost threefold.

CDC supports many evidence-based public health interventions. Their National Comprehensive Cancer Control Program (NCCCP; www.cdc.gov/cancer/ncccp/) funds every US state, territory, and tribe or tribal organization to develop and implement evidence-based plans to control cancer. CDC recommends that state comprehensive cancer control plans include evidence-based recommendations and guidelines, such as those from the *Community Guide* and the USPSTF. These interventions include patient reminders, reducing structural barriers, provider reminders, provider assessment and feedback, small media programs, one-on-one education for cancer screening, multicomponent interventions, and interventions that engage community health workers (31). The inclusion of evidence-based interventions in cancer control plans is an area for improvement. Soori and colleagues evaluated current comprehensive cancer control plans for 50 states and the District of Columbia for inclusion of evidence-based breast cancer control recommendations and guidelines (33). They found that only 6% to 37% of plans included USPSTF recommendations for breast cancer interventions, and only about half included mammogram prevalence in the burden statement. A previous mixed-method study done by CDC found that developers of comprehensive cancer control programs were familiar with evidence-based interventions but needed assistance in implementing them and evaluating their success (34).

Increasing cancer screening will require the collective effort of policy makers, public health practitioners, researchers, and primary care providers. Using evidence-based, multicomponent interventions can increase screening among populations with low screening rates (35). Culturally tailored strategies could be developed to address the needs of socioeconomically disadvantaged and medically underserved groups (29,36). Research and evaluations of public health programs need to focus on the roots of barriers and develop innovative strategies to increase screening. Factors that affect cancer screening behaviors are intertwined. Resolving just one will not solve the whole screening issue. For example, cancer screening rates are generally low among people with low incomes or who lack health insurance (37,38). However, offering health insurance to the uninsured may not be sufficient to increase rates. Medicaid beneficiaries have health coverage for cancer screening, but they may not be able to afford the cost of transportation or loss of a day’s pay for a colonoscopy (31,39–41). The financial burden associated with transportation and loss of work should be considered and evaluated. Developing innovative cancer screening techniques that are portable, noninvasive, and low cost could also increase the uptake of cancer screening.

The ultimate goal of cancer screening is to reduce cancer incidence and mortality (36). Thus, cancer screening can be coupled with primary cancer prevention strategies to reduce cancer risks and to increase proper follow-up care and treatment, especially with the ongoing COVID pandemic in which preventive medical procedures and tests may be delayed or postponed. Public health needs to build the infrastructure to be better prepared so that cancer education, screening, and early treatment are minimally affected by the next pandemic, thereby saving lives.
